# Some Remarks on Angina Pectoris

**Published:** 1784

**Authors:** 


### SECTION II.

### Essays and Observations.

I. *Some Remarks on Angina Pectoris, communicated in a letter to* Samuel Foart Simmons, M. D. F. R. S. *by* Malcolm Macqueen, M. D. *physician at Yarmouth.*

IT was my opinion, even before I left the university, that the disease described by Dr. Heberden and the late Dr. Fothergill, under the name of *Angina Pectoris*, had been, in the greater number of instances, if not in all, an irregular gout. The sex and habits of the patients, the form of their bodies, and their time of life, correspond with the notion we have of the gouty diathesis. The flatulent eructations from the stomach, the benefit frequently received from aromatics and Bath waters, &c. also lead to the same

ſame concluſion. But the following caſe proves beyond doubt, that the diſeaſe is in ſome inſtances intimately connected with gout.

About twelve months ago I was called to a man 60 years of age, of a fair, ſanguine complexion, and a ſtrong and corpulent habit, with a ſhort neck. He complained of frequent fits of ſudden pain about the pit of the ſtomach, extending to the left breaſt, and ſhooting towards the middle of both arms, particularly the left. This pain or ſpaſm was accompanied with a difficulty of breathing that threatened ſuffocation. It had ſeized him for ſome time before, and often in his ſleep; but he was more peculiarly ſubject to its attack when he attempted to walk quick, or againſt a ſtrong wind; he was then obliged to ſtop ſhort in an inſtant, and remain at perfect reſt for one or two minutes, in which time the diſorder generally went off. He obſerved alſo, that the fit was apt to return by the leaſt exciting cauſe ſoon after meals; inſomuch indeed that he uſed to ſay, "he ſhould be well, "if he could live without food." Paſſion too, or any ſtrong agitation of mind, ſeldom failed to bring it on. His pulſe was pretty regular, his bowels not coſtive, yet his appetite was very indifferent, and he complained much of flatulency.

Such was the ſtate I found him in, and he then gave me the following account:

He ſaid that he had always been accuſtomed to live well, and for ſeveral years had been ſubject to regular fits of the gout in his feet. But about ſix months before, when his feet were ſlightly affected, he perceived a general faintneſs, and a dizzineſs of his head, which recurred frequently. He applied to his apothecary, who immediately bled him, and inſerted an iſſue in his left arm. Very ſoon after this the gout entirely left his feet, and he felt the firſt attack of the pain and ſtricture acroſs the præcordium. This new complaint returned by ſhort and ſlight fits, which grew gradually ſtronger till the time I ſaw him, and from its firſt acceſſion he had been free from any uneaſineſs of his feet, and every other ſymptom of the regular gout.— From this hiſtory of his diſorder, and the conſideration of the preſent ſymptoms, I did not heſitate in imputing the whole to a gouty affection: accordingly he was ordered aromatic bitters in conſiderable quantity, and a draught with *tinct. guaiac. vol.* twice a day. By theſe medicines and a ſuitable regimen he was ſoon conſiderably relieved; and I left him on this plan, hoping to hear in a ſhort time that he had got a regular regular fit of the gout. In this wiſh, however, I was diſappointed, and, in about two weeks after, he was ſeized, in the night-time, with a moſt violent paroxyſm of the diſorder in his ſtomach and breaſt. It continued four-and-twenty hours with ſcarce any intermiſſion, before I ſaw him, notwithſtanding he had taken freely of ſpirituous bitters and *tinct. guaiac. vol.*—I immediately ordered a draught of *tinct. fœtid. vol.* ℥ß. and *elixir. paregor.* ʒj. without any other dilution. This draught, which he imagined had burned his whole inſide, removed his complaint almoſt inſtantaneouſly. A warm plaiſter was applied to the pit of his ſtomach, and his feet were bathed in warm water largely impregnated with common ſalt. He felt no further affection of his ſtomach or breathing, and a fit of the gout in the right foot ſucceeded on the enſuing night. This fit fortunately proved a ſmart and a long one; for he was confined to his houſe upwards of three months, during which time he had not the ſlighteſt return of his former complaints. I have ſeen him within theſe few days, apparently in good health. He ſays, that in the intervals of the gout he is ſtill ſubject to ſlight degrees of his diſorder, when he attempts to walk quick after after dinner; but is perfectly free from it in bed, and when at reſt in the day-time.

The above caſe, which is taken from my Journal, proves, I think, beyond a doubt, that the *angina pectoris* may ariſe from an irregular gout. That it does ſo in many inſtances, I have always thought highly probable: to aſſert that it does ſo in all, might appear too bold.

Diſſections ſeldom have thrown any light on the nature of the diſeaſe, except indeed in a negative way, by ſhowing us, from the want of any uncommon appearance, that it is a ſpaſmodic affection. Dr. Fothergill himſelf confeſſes, that it terminated for the moſt part fatally; and though it was a diſeaſe he frequently met with, yet it was not in his power, in a ſingle inſtance, to learn the ſeat of it from diſſection. Altho' in ſome few caſes, water has been found in the thorax, this is to be conſidered as an effect only of the diſeaſe. Water in the cheſt is often a conſequence of ſpaſmodic aſthma, and may alſo follow an angina pectoris, which bears a ſtrong analogy to the aſthma, and in the judgment of ſome, ought to be conſidered as one and the ſame diſeaſe.

It might be thought too confident an aſſertion, to ſay, that the ſtomach is the organ chiefly and primarily affected here. It cannot be denied, however, that in this diſorder the ſtomach is invariably affected to a certain degree. Dr. Heberden remarks, " that the patients feel the " ſtricture under the ſternum, and always to- " wards the left ſide." My patient felt the ſtricture about the *cartilago enſiformis*; and in one of the caſes related by Dr. Fothergill, the villous coat of the ſtomach was found to be inflamed after death.—In the ſpaſmodic aſthma, the paroxyſm is always attended with flatulency at the ſtomach: ſubſtances that cauſe this flatulency, and that otherwiſe diſagree with the ſtomach, frequently bring on the paroxyſm. In like manner, different remedies taken into the ſtomach ſometimes very quickly remove it. Why may not a like ſympathy take place betwixt the ſtomach and lungs in the angina pectoris as in aſthma? I own I think the connection ſtill greater in the former.

I am well aware, that this is not a new idea; and that the two learned gentlemen mentioned above have adminiſtered aromatics and ſtimulants, probably on the ſuppoſition of its being a gouty affection of the ſtomach. Yet their want of ſucceſs does not prove the diſeaſe to be unconnected with the gout; or that aromatic and and ſtimulant medicines, differently doſed and qualified, might not in many inſtances, as in that of my patient, have a better effect, and prove more ſucceſsful than the iſſues in the thighs, which I know are found to fail in a great number of caſes, if not in all.

I have another caſe before my eye, that I think deſerves to be mentioned here, from the analogy it bears to the former.

About three years ago, I was called to a gentleman of this town, who was then paſt 50 years of age, of a full habit of body and a florid complexion. He had always enjoyed good health, till within three or four months, that he had become ſubject to frequent returns of pain about his heart, as he termed it, pointing to the *cartilago enſiformis* towards the left ſide. This pain, which had from its beginning increaſed in frequency and violence, was conſtantly attended with another pain, and a ſenſe of tenſion in the right fore arm, extending along the aponeuroſis of the biceps to the wriſt, and cauſing, for the time, little knots in the fleſh, like partial contractions of muſcular fibres. The affection of the breaſt and that of the right forearm conſtantly accompanied each other, the latter immediately ſucceeding the former, and likewiſe wiſe diſappearing along with it. Any exertion, as quick walking, or agitation of ſpirits, was apt to bring on the diſorder; but it was certain to return every night, ſometimes twice, ſometimes three times before the morning. It attacked him both awake and aſleep, and he was always forced to raiſe himſelf from an horizontal to an erect poſture, while the fit laſted, which was generally from half an hour to a full hour; and it always went away by an eructation of wind from the ſtomach.

After I had conſidered the ſymptoms of this caſe, I was ſtrongly of opinion, that it would terminate either in a regular gout, or in angina pectoris, and might poſſibly partake of both. I have, however, been hitherto diſappointed.— The complaint is greatly relieved, I might ſay almoſt cured, without aſſuming the character of either of the above diſeaſes in any regular form. After adminiſtering a few anodynes at firſt, by way of experiment, and obſerving no manifeſt benefit from them, I continued afterwards to treat it as irregular gout. The chief remedies employed were boluſes of prepared ſteel and cinnamon given too or three times a day, and waſhed down with a draught of ſtrong peppermint water, a warm plaiſter to the re-

 gion gion of the ſtomach, and a ſuitable regimen, particularly an abſtinence from acids, and all flatulent food. Under this plan he ſoon began to amend, and the diſorder, though not wholly eradicated, became ſo ſlight as to give ſcarce any uneaſineſs. The next year, the ſame ſymptoms returned with ſome violence, and happening to be in a neighbouring city, he conſulted a phyſician of great experience and deſerved reputation. He, I believe, conceived an idea different from mine of the diſeaſe, and the patient was ordered to loſe ℥xij. of blood, and to take afterwards ſome purges of calomel. This was complied with on his return home, without the leaſt apparent benefit, but rather a contrary effect. He then had recourſe to his former medicines, which proved equally ſerviceable as they had done the year before. He has ever ſince attended carefully to the regimen I had at firſt laid down for him, takes one of his boluſes every day, with the addition of a little bark, and continues almoſt intirely free from every ſymptom of his diſorder.

To thoſe who admit the Cullenian doctrine of the gout, particularly what is ſet forth in the ſection on the atonic gout, in Dr. Cullen's Firſt Lines of the Practice of Phyſic, my ſentiments conconcerning the two foregoing cafes will probably appear rational and confiftent. But, independent of all fyftems, we are convinced by experience and obfervation, that the ftomach is peculiarly difpofed to be affected in gout; that it is fubject to atony and fpafms, which are alfo frequently communicated to other parts that fympathize with that organ. The firft of the two cafes, if I am not miftaken, affords one inftance at leaft of the angina pectoris being intimately combined with gout, and, according to my opinion, appearing as a form of irregular gout. If the fecond fhall appear to you not to partake of the character of gout, ftill it will ferve to fhew a curious fympathy exifting betwixt the ftomach and the fore-arm, not unlike what takes place in the angina pectoris, and this removed by aromatic medicines applied to the ftomach.—We have yet reafoned a fhort way only on the doctrine of fympathy, facts on this head are of courfe the more valuable; and the prefent is the age of facts and obfervation.

Yarmouth, Apr. 8,
1784.

